# Supplementary material for: Can big data increase our knowledge of local rental markets? A dataset on the rental sector in France
Source: PLoS One. 2022 Jan 27;17(1):e0260405. doi: 10.1371/journal.pone.0260405 (PMC8794157; doi:10.1371/journal.pone.0260405)

S.1 Table. Comparison between the median rent provided by online ads and the OLL, following the market tightness

	(1)	(2)	(3)	(4)	(5)	(6)
	Below Median			Above Median		
Ads	0.0288	0.0288	0.0288	0.0548	0.0548	0.0548
	(0.0639)	(0.0699)	(0.0639)	(0.0957)	(0.113)	(0.0957)
N	334	334	334	404	404	404
R2	0.000	0.753	0.944	0.000	0.712	0.942
N. rooms	N	Y	N	N	Y	N
Area FE	N	Y	N	N	Y	N
Area FE x N. rooms	N	N	Y	N	N	Y

Standard errors in parentheses clustered at the Agglomeration levels and at the dataset level
* $p < 0.05$, ** $p < 0.01$, *** $p < 0.001$

Note: Estimates of $Rent_{d,a,r} = \alpha + \beta \times 1_{d=Ads} + X_{a,r}\lambda$. N. Rooms corresponds to the inclusion of rooms fixed effect (1,2,3,4+). Area FE corresponds to the inclusion of area fixed effects. Sampling areas are defined by OLL and mostly cover groups of municipalities. Area FE x N. rooms corresponds to the inclusion of interaction terms between Area and N. rooms.

S.2 Table. Hedonic models of housing price and rent, for one department

	Dependent variable:					
	Appartments		Single Units		All	
	(1)	(2)	(3)	(4)	(5)	(6)
	ln(price/surf)	ln(rent/surf)	ln(price/surf)	ln(rent/surf)	ln(price/surf)	ln(rent/surf)
Constant	7.40***	2.28***	7.76***	2.39***	7.33***	2.28***
	(0.02)	(0.01)	(0.02)	(0.01)	(0.03)	(0.01)
Surface (log)	-0.34***	-1.18***	-0.76**	-0.13	-1.22***	-1.26***
	(0.13)	(0.06)	(0.35)	(0.25)	(0.23)	(0.08)
Surface (log)^2	0.02	0.09***	0.06*	-0.03	0.14***	0.10***
	(0.02)	(0.01)	(0.04)	(0.03)	(0.03)	(0.01)
1 Room (Ref. 4 Rooms)	-0.03	-0.10***	-0.14	-0.23***	0.03	-0.09***
	(0.03)	(0.01)	(0.09)	(0.07)	(0.04)	(0.02)
2 Rooms (Ref. 4 Rooms)	0.02	-0.05***	-0.07	-0.14***	0.09**	-0.05***
	(0.02)	(0.01)	(0.04)	(0.04)	(0.04)	(0.01)
3 Rooms (Ref. 4 Rooms)	0.03**	-0.02***	-0.03*	-0.07***	0.07***	-0.02**
	(0.01)	(0.01)	(0.02)	(0.02)	(0.02)	(0.01)
5 Rooms or more (Ref. 4 Rooms)	-0.01	0.03***	-0.01	0.01	-0.05**	0.02
	(0.01)	(0.00)	(0.01)	(0.01)	(0.03)	(0.01)
R2	0.63	0.77	0.65	0.70	0.62	0.77
N	8266	31793	3340	3890	4942	27903

*p<0.1; **p<0.05; ***p<0.01

S.1 Fig. Distribution of the rent-price ratio by municipalities

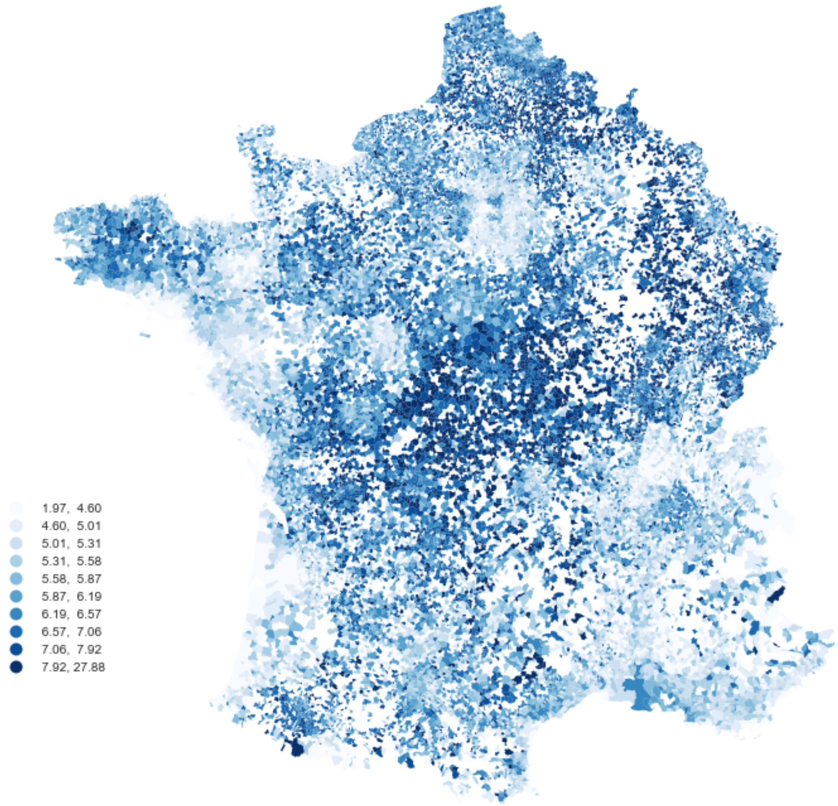

Note: Price data are taken from fiscal administrative database *Demande de Valeur Foncière*, rent data are taken from the database constructed by the authors. The rent-price ratio is calculated as the ratio of municipal hedonic price index and hedonic rental index. Author's computations and ADMIN EXPRESS COMMUNE which is under an Open Licence Etalab `https://www.data.gouv.fr/fr/datasets/admin-express/`

S.3 Table. Parameters of the rent ceiling in the social housing sector

	PLA-I	PLUS	PLS	PLI
Subsidized interest rate (with respect to livret A)				
	-0.2pp	+0.6 pp	+1.1 pp	+1.4 pp
Other State Subsidies				
Brick and Mortar	<20%	<5%	No	No
Reduced VAT	Yes	Yes	Yes	No
Property Tax exemption (25 years)	Yes	Yes	Yes	No
Local Subsidies				
loan guaranty	Yes	Yes	Yes	No
Maximum Rent Per square meter				
Ibis (Paris for PLI)	5.42	6.09	9.14	16.82
I (A for PLI)	5.09	5.73	8.60	14.01
II (B for PLI)	4.46	5.03	7.54	9.74
III (C for PLI)	4.14	4.67	7.01	7.01
Share of households eligible				
	30%	65.5%	80.7%	87.4%

S.4 Table. Determinants of the rent-price ratio

	Rent/price	Rent/price
2 Rooms (Ref. 1 Room)	-0.83***	-0.95***
	0.01	0.027
3 Rooms (Ref. 1 Room)	-1.2***	-1.46***
	0.009	0.037
4 Rooms (Ref. 1 Room)	-1.53***	-1.73***
	0.009	0.041
5 or more Rooms (Ref. 1 Room)	-1.7***	-1.85***
	0.01	0.042
Type: house	0.04***	-0.79***
	0.009	0.037
Municipality FE	N	Y
R2	0.12	0.55
N	714437	714437

*p<0.1; **p<0.05; ***p<0.01

S.5 Table. Determinants of the dwelling level benefit per square meter

	(1) ln(subsidy)	(2) ln(subsidy)	(3) ln(subsidy)
2 Rooms (ref. 1 Room)	-0.298***	-0.170***	-0.140***
	(0.0341)	(0.0122)	(0.0147)
3 Rooms (ref. 1 Room)	-0.482***	-0.297***	-0.254***
	(0.0387)	(0.0271)	(0.0318)
4 Rooms (ref. 1 Room)	-0.584***	-0.359***	-0.303***
	(0.0449)	(0.0384)	(0.0431)
5+ Rooms (ref. 1 Room)	-0.618***	-0.392***	-0.329***
	(0.0430)	(0.0492)	(0.0528)
PLI (ref. PLAI)	-0.150	-0.528***	-0.591***
	(0.193)	(0.0953)	(0.0837)
PLS (ref. PLAI)	-0.157	-0.476***	-0.554***
	(0.142)	(0.0510)	(0.0640)
PLUS (ref. PLAI)	-0.184***	-0.212***	-0.187***
	(0.0498)	(0.0321)	(0.0236)
1950-1959 (ref. 1900-1949)	-0.0527	0.181**	0.137***
	(0.139)	(0.0746)	(0.0151)
1960-1979 (ref. 1900-1949)	-0.197	0.127	0.0856***
	(0.182)	(0.0949)	(0.0133)
1980-1999 (ref. 1900-1949)	-0.529***	-0.176***	-0.225***
	(0.136)	(0.0546)	(0.0420)
After 2000 (ref. 1900-1949)	-0.568***	-0.130*	-0.211***
	(0.203)	(0.0709)	(0.0320)
Before 1900 (ref. 1900-1949)	-0.258***	-0.0364	-0.102***
	(0.0660)	(0.0405)	(0.0284)
Municipality FE	N	N	Y
Social housing area FE	N	Y	N
R2	0.11	0.60	0.83
N	4404351	4404351	4404351

Standard errors in parentheses

* $p < 0.10$, ** $p < 0.05$, *** $p < 0.01$

S.6 Table. Determinants of the city level benefit per square meter

	(1)	(2)	(3)	(4)
	ln(subsidy)	ln(subsidy)	ln(subsidy)	ln(subsidy)
Panel A) Subsidy and private rent level				
ln(rent)	2.443***	2.431***	2.376***	2.827***
	(0.019)	(0.019)	(0.021)	(0.058)
ln(pop)		0.005	0.009**	0.01*
		(0.003)	(0.003)	(0.005)
% Empty			-0.7***	-0.136
			(0.122)	(0.155)
R2	0.78	0.78	0.78	0.76
N	8542	8542	8542	8542
Panel B) Subsidy and Social tenants income				
ln(social income)	0.865***	0.767***	0.476***	0.22***
	(0.039)	(0.036)	(0.037)	(0.034)
ln(pop)		0.273***	0.257***	0.103***
		(0.006)	(0.005)	(0.006)
% Empty			-5.807***	-2.867***
			(0.175)	(0.191)
R2	0.07	0.27	0.36	0.25
N	8542	8542	8542	8542
Urban unit FE	N	N	N	Y

* $p < 0.10$, ** $p < 0.05$, *** $p < 0.01$

Note: The dependant variable is the municipal fixed effects obtained from the regression described in column (3) in Table S.5. These can be interpreted as an index for a representative social dwelling corresponding to a flat with 1 room financed by a PLAI and built between 1900 and 19149

S.7 Table. Descriptive statistics on the restricted sample dropping outliers in price per square meter

	Count	Mean	Std	Min	25%	50%	75%	Max
Gross Rent	3933308.0	636.2	334.0	85.0	448.0	565.0	730.0	13000.0
Surface	3933308.0	53.5	27.4	6.0	34.0	49.0	67.7	490.0
Gross Rent per square meter	3933308.0	13.1	5.0	6.2	9.3	11.9	15.8	29.1
Time elapsed since publication (days)	3933308.0	29.6	39.4	0.0	8.0	20.0	38.0	770.0
Expenditures (%): Included	3933308.0	72.9	44.5	0.0	0.0	100.0	100.0	100.0
Expenditures (%): Not Included	3933308.0	5.4	22.5	0.0	0.0	0.0	0.0	100.0
Expenditures (%): Unknown	3933308.0	21.7	41.2	0.0	0.0	0.0	0.0	100.0
Amount Expenditures	587904.0	60.8	53.9	0.0	30.0	48.0	80.0	3380.0
Collective heating (%)	3933308.0	3.6	18.6	0.0	0.0	0.0	0.0	100.0
Hot water (%)	3933308.0	0.2	4.2	0.0	0.0	0.0	0.0	100.0
Trash collection (%)	3933308.0	4.7	21.1	0.0	0.0	0.0	0.0	100.0
Furnished (%): No	3933308.0	76.9	42.2	0.0	100.0	100.0	100.0	100.0
Furnished (%): Yes	3933308.0	23.1	42.2	0.0	0.0	0.0	0.0	100.0

S.2 Fig. Number of ads and vacancy

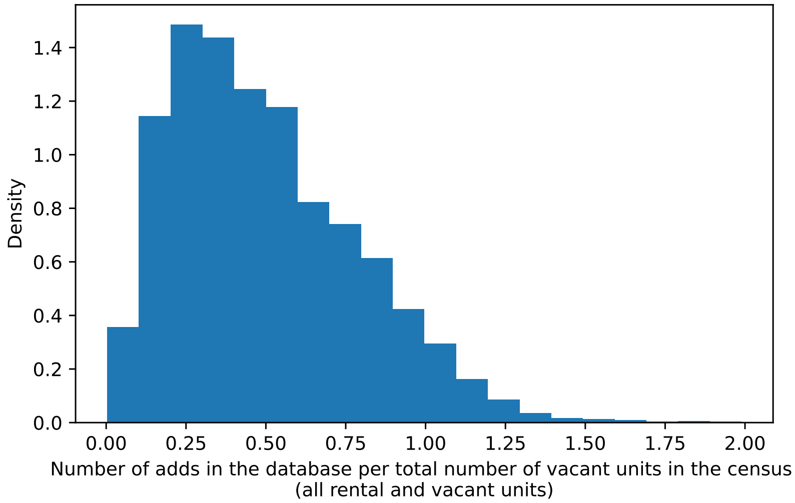

Supplement: S1 Appendix — (PDF) [file pone.0260405.s001.pdf]
