# Supplementary figures and images for: Can big data increase our knowledge of local rental markets? A dataset on the rental sector in France
Source: PLoS One. 2022 Jan 27;17(1):e0260405. doi: 10.1371/journal.pone.0260405 (PMC8794157; doi:10.1371/journal.pone.0260405)

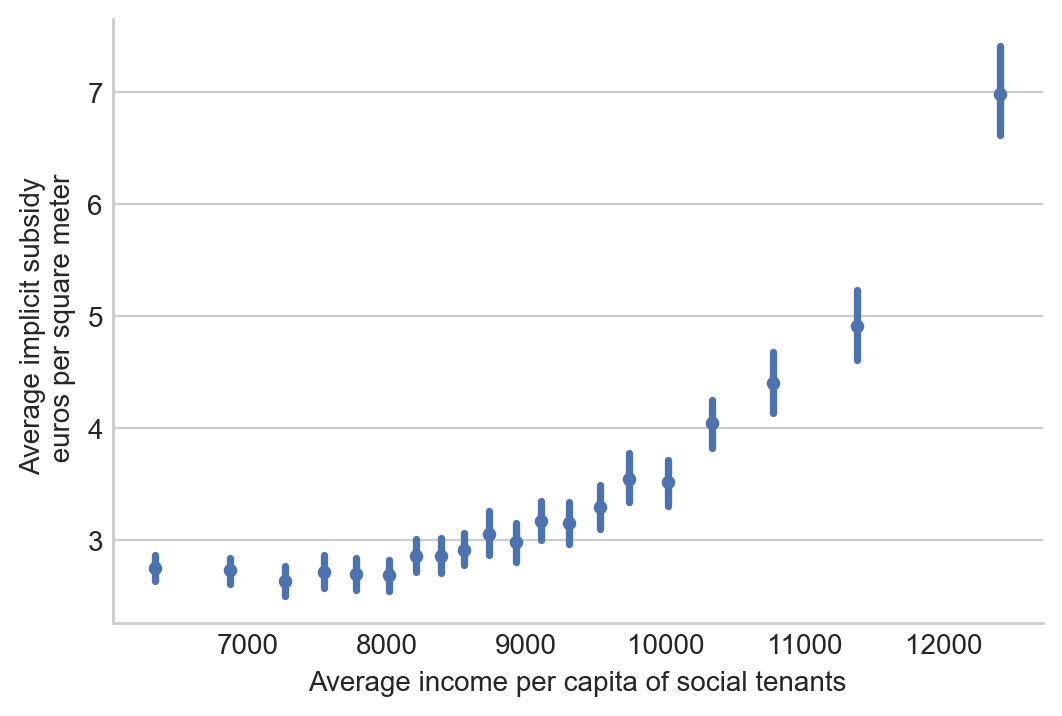

Supplement: S1 File — (ZIP) [file pone.0260405.s002.zip › charts/average_subsidy_income.png]

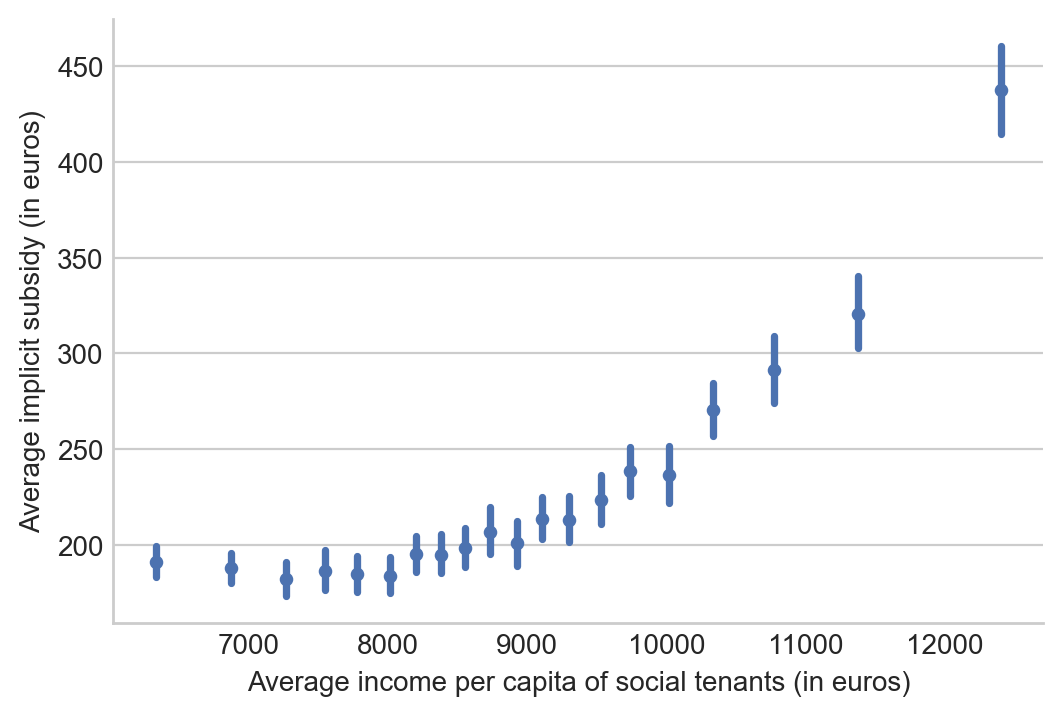

Supplement: S1 File — (ZIP) [file pone.0260405.s002.zip › charts/average_subsidy_income_level.png]

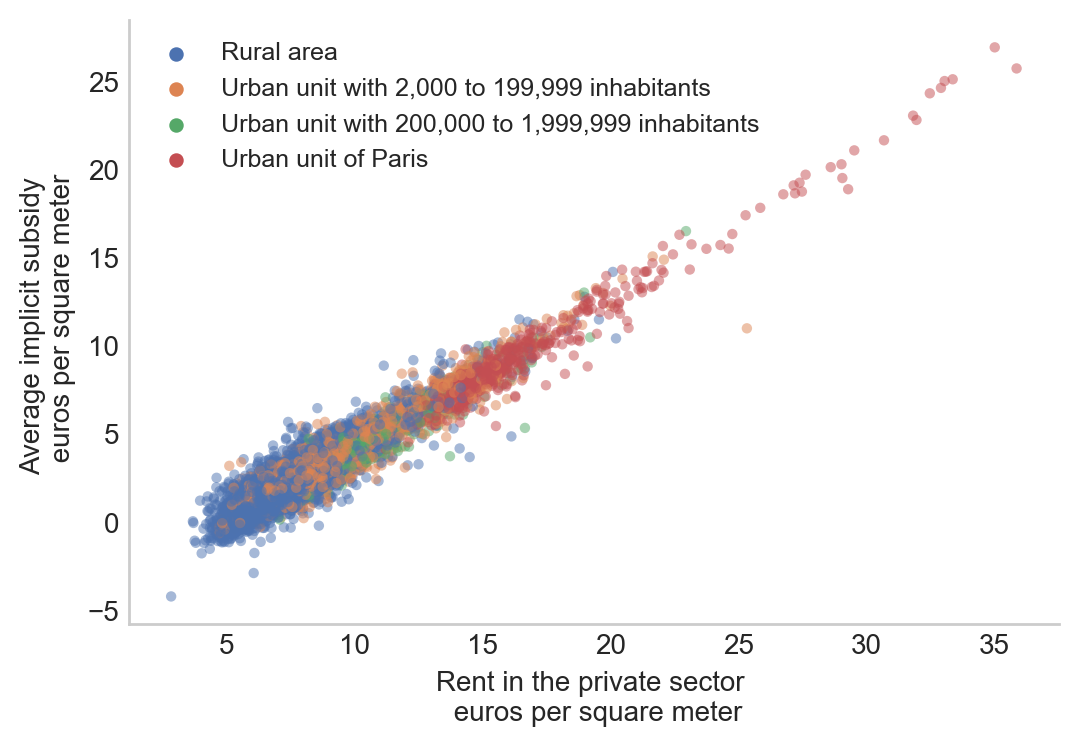

Supplement: S1 File — (ZIP) [file pone.0260405.s002.zip › charts/average_subsidy_municipal.png]

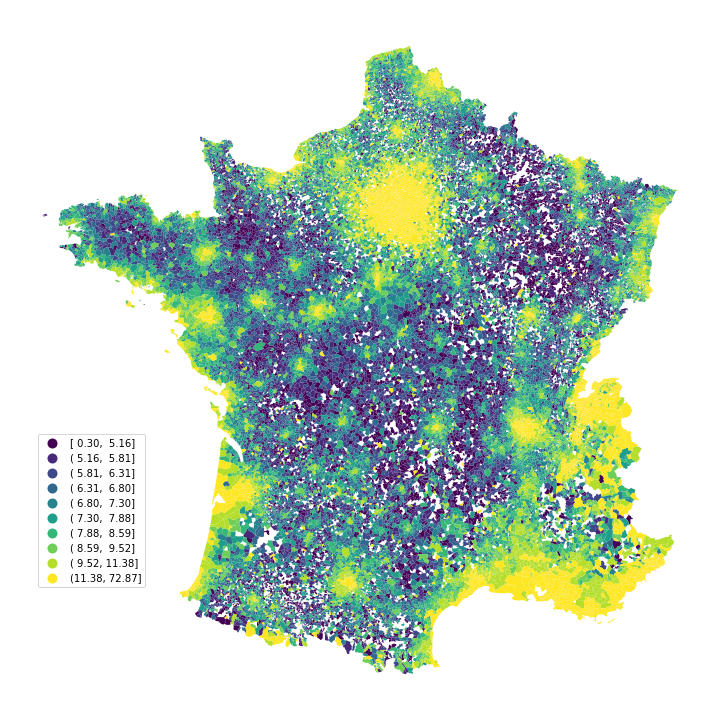

Supplement: S1 File — (ZIP) [file pone.0260405.s002.zip › charts/Average-France.png]

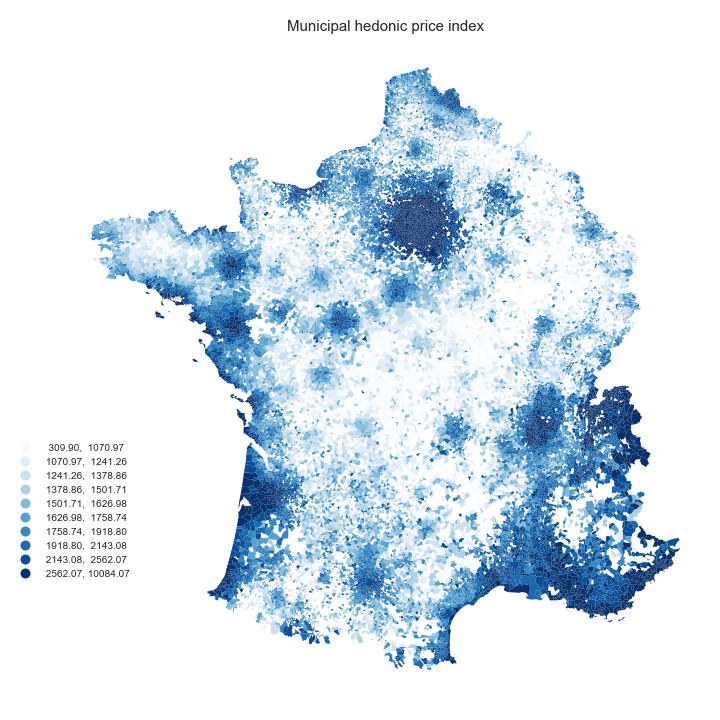

Supplement: S1 File — (ZIP) [file pone.0260405.s002.zip › charts/Map_France_price.png]

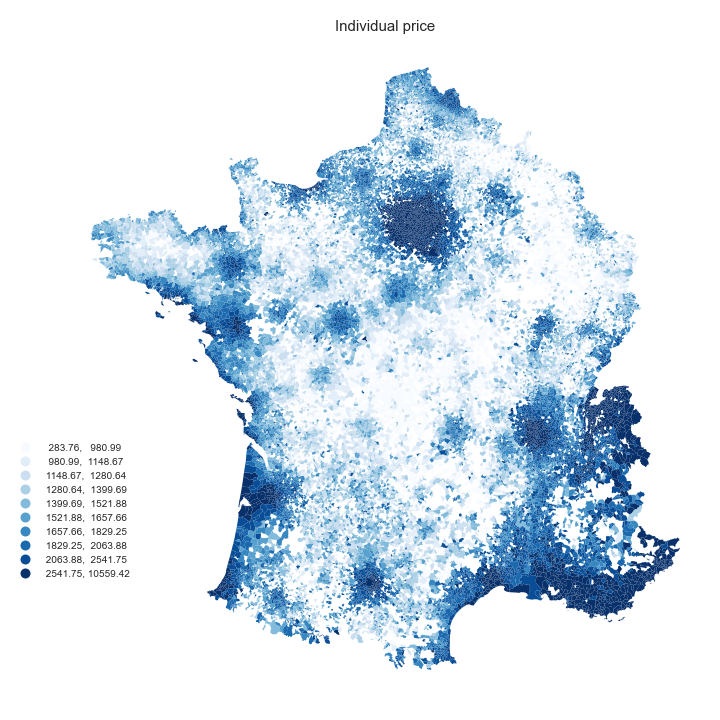

Supplement: S1 File — (ZIP) [file pone.0260405.s002.zip › charts/Map_France_price_ind.png]

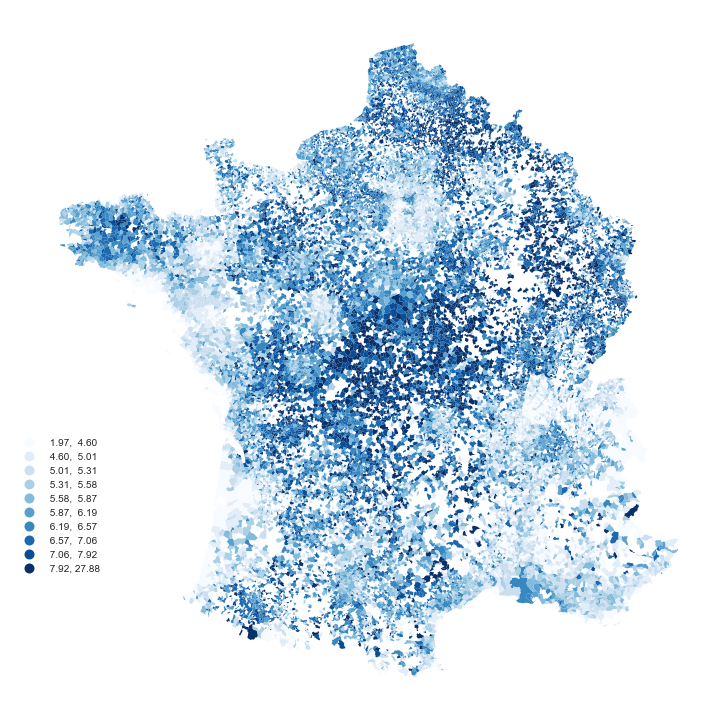

Supplement: S1 File — (ZIP) [file pone.0260405.s002.zip › charts/Map_France_rent_price.png]

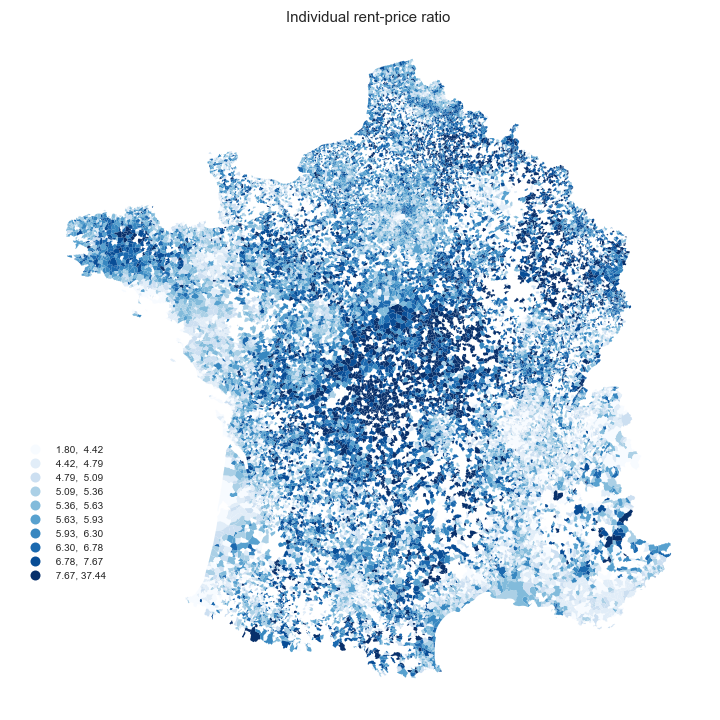

Supplement: S1 File — (ZIP) [file pone.0260405.s002.zip › charts/Map_France_rent_price_ind.png]

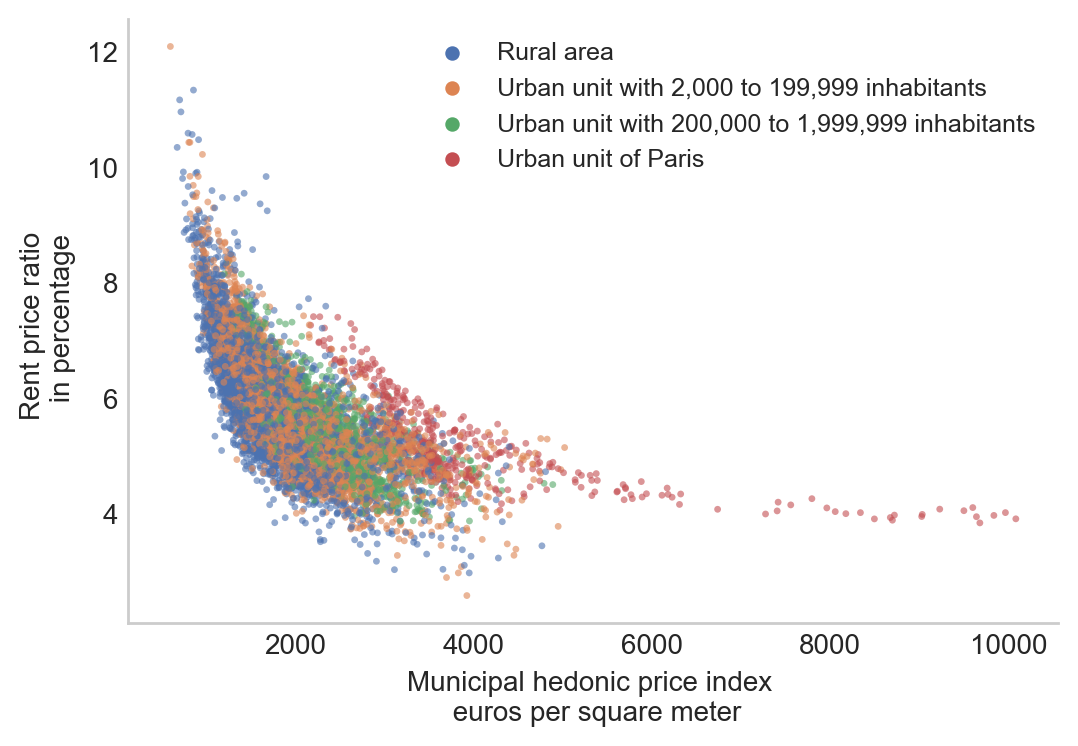

Supplement: S1 File — (ZIP) [file pone.0260405.s002.zip › charts/municipalities_rent_price_ratio.png]
