# Supplementary material for: Can big data increase our knowledge of local rental markets? A dataset on the rental sector in France
Source: PLoS One. 2022 Jan 27;17(1):e0260405. doi: 10.1371/journal.pone.0260405 (PMC8794157; doi:10.1371/journal.pone.0260405)
Supplement: Attachment — Submitted filename: ReviewPlosOne.pdf [file pone.0260405.s003.pdf]

Title:

Can big data increase our knowledge of local rental markets? a dataset on the rental sector in France

Paper summary:

The authors use web-scraping technology to create a dataset of French rental properties, including detailed property characteristics and asking rents. They present descriptive statistics of the dataset and use existing, smaller datasets to show that the data are unbiased and reflecting local rent markets accurately. Using web-scraping can therefore be seen as a low-cost way to create large and detailed datasets of rental properties in France.

Feedback summary

Overall, the authors did a great job creating this extensive dataset. However, even though the presented methodology is advertised as low-cost, potential obstacles and the required effort should be better discussed. The authors had to scrape the websites on a monthly base for around 2 years (l. 105) and used cooperative websites, providing public APIs (l.100). This effort might not be suitable for some purposes, such as when needing timely data or historical data. Furthermore, many similar websites in other countries are less cooperative, employing software to actively prevent scraping.[1]. However, my main problem with the study is the lack of originality in its current form. As explained below, there are commercial software solutions for web-scrapping technology. Furthermore, other studies have already explored scrapped real estate data or use them actively to answer other research questions. The authors discuss potential applications using "better", scrapped data. Maybe some of these could be further explored to set the study apart from similar studies.

Major Issues:

- The authors claim that their methodology could be used in other countries but don't discuss potential obstacles. Since the methodology is at the center of the study, the pros and cons should be discussed in

[1]See an example solution with a list of clients here `www.datadome.co`

more detail. Potential disadvantages could be real estate websites do not provide a public APIs and even try to prevent web scraping using software, different legal situations (e.g. copyright laws or terms & agreements of websites), and the lack of historical data.

- One major assumption is the relation between asking rents and contracted rents. The authors argue that France is a seller's market with lack of housing supply (l.219). Therefore asking and contracted rents should be similar. However, this could be very different over real estate cycles and in other markets. It would be interesting if the authors could discuss this a little bit more and explore some local heterogeneity (e.g. regions with more and less housing supply).

- By using the existing rent data as a benchmark, the authors put themselves in a difficult situation to show the superiority of the new data (except for coverage). Since the cost of alternative datasets are not discussed, a main argument for using scrapped data could be the richness in controls. The authors should therefore highlight the advantages more (besides potential costs savings and higher coverage). Maybe it could help to assume that the comparison data are not the benchmark, but inferior in terms of quality (e.g. some are survey based).

- My main criticism is that the authors should expand their study to increase their contribution. Using web-scraping itself is already available in software packages.[2] Furthermore, web-scrapping has already been used and explored in a real estate context [3, 4] and the reliability has been investigated [1, 2] (even for France). Therefore, instead of focusing on descriptive statistics, comparing to inferior data, the authors should focus on possible use cases and advantages of the dataset. Some ideas could be to explore better hedonic characteristics, spatial heterogeneity, estimating new indexes, show implications for tax purposes (as the authors suggest in the introduction), or strengthen the cost argument.

Minor Issues:

- The description of the methodology is very detailed, but the authors do not state the names of scrapped websites. They only mention that

[2] `https://www.octoparse.com/blog/top-30-free-web-scraping-software`

these are the wo largest (l. 87). For transparency the websites should be named.

- Figure 1 is a bit confusing and a bit misleading. I suggest to remove the software and programming languages from it, since web-scarping could be done in many different ways and HTML code is not something that is scraped here. In line 101 the authors talk about a public API, which is not in the diagram and suggests a slightly different process.
- The quartile estimates in Figure 5 add not too much value since they are not readable for most observations. Maybe drop them for simplicity.
- There are a several grammar and spelling mistakes (l. 279 "on-line"). I suggest a professional proofreading service.

References

[1] G. Boeing and P. Waddell. New insights into rental housing markets across the united states: Web scraping and analyzing craigslist rental listings. *Journal of Planning Education and Research*, 37(4):457–476, 2017.

[2] G. Boulay, D. Blanke, L. Casanova Enault, and A. Granié. Moving from market opacity to methodological opacity: Are web data good enough for french property market monitoring? *The Professional Geographer*, 73(1):115–130, 2021.

[3] M. Gindelsky, J. Moulton, and S. A. Wentland. Valuing housing services in the era of big data: A user cost approach leveraging zillow microdata. In *Big Data for 21st Century Economic Statistics*. University of Chicago Press, 2019.

[4] M. Loberto, A. Luciani, and M. Pangallo. What do online listings tell us about the housing market? *Updated version of Bank of Italy Temi di Discussione (Working Paper) No*, 1171, 2020.
