# Supplementary material for: Can big data increase our knowledge of local rental markets? A dataset on the rental sector in France
Source: PLoS One. 2022 Jan 27;17(1):e0260405. doi: 10.1371/journal.pone.0260405 (PMC8794157; doi:10.1371/journal.pone.0260405)
Supplement: Attachment — Submitted filename: Response_referee_2.pdf [file pone.0260405.s004.pdf]

3 Referee 2

The purpose of this short paper is to present a novel database of housing rents in France. As is the case in many other countries, there exist few easily accessible databases of rental prices. The authors scrap data from two major real estate websites to obtain a large database of 4.3 million housing rents in France, covering the period from December 2015 until January 2018. The authors provide descriptive statistics and examine the representativeness of their database by comparing it to other databases.

From my reading of the paper, the data collection seems to have been executed properly and the resulting database could help us to acquire some new knowledge on the functioning of rental markets, in particular in France.

I provide my main comments and suggestions to this study below.

Major comments:

1. From the manuscript it does not become clear where the data will be published. The manuscript replicates the sample text "ALL XXX files are available in the Open Science Framework Repository (accession number(s) XXX, XXX.)", but did not replace the XXX with the right information. At the OSF Repository, I was unable to find the database using the paper title. It is also not explicitly indicated that data will only be published at a later stage.

   Sorry for the confusion. Here we just followed the guideline of PLOS ONE which allows to provide the link to the dataset after the acceptation.

2. There are quite a few typos and grammatical errors in the manuscript, and I would suggest having the manuscript read and verified by a native copy editor. I am not a native speaker myself, but at the bottom of this review, I have provided a few examples from the first page. I have ignored them later in the manuscript because this would make my review lengthy.

   Sorry for this. We submitted this new version to an english editing service.

Minor comments:

3. In the opening lines of the paper, the authors argue that historically the French authorities have recorded housing transactions and expressed limited interest in recording rental prices. I do not fully agree with this statement. For extended periods of time (at least until the mid-20th century) the French fiscal administration has been keeping enormous registrations of rental contracts, for example in the Enregistrement, since many taxes were based on rental prices rather than sales prices.

   Thanks for raising this point. We removed this assertion to account for your comment

4. In the tables, the Min / 25% / 50% / 75% / Max values generally do not seem to add much, since most of the variables that are presented

are dummy variables rather than continuous variables. I would suggest removing these statistics.

We adjusted the tables where all variables are dummies.

5. It would be useful to briefly mention the actual websites and companies that were used to collect the data and to provide some statistics on their market share / user base.

   We do it now and discuss their market share.

6. I would suggest adding some references to comparable work in other countries. Most notably, Boeing Waddell (2016, Journal of Planning Education and Research) have scraped data from Craigslist in a very similar fashion.

   We are aware of this paper which was already quoted. As the references are numbered it might be harder to see where it is. We quote it now earlier in the paper. This is the reference number 15.

7. For the representativeness of the database, it seems important to also consider the presence of social housing (HLM and other types), which are likely reported in the census but, based on what I assume, will not be published on these online websites.

   Sorry, we were not precise on this. We only account for private rental sector and remove HLM in our analysis. We specify this more precisely now.

8. The section about the comparison to the French census is at times confusing. For example, in line 190: "In a second step we assign our posted scraped to each strata". It is not clear to me what "posted" is (it might be a typo). In line 193 it is unclear what "goods" refers to.

   We meant "ad". Sorry for this.

   Some example typos from the first page:

   Line 26: as the transaction is taxed instead of "when"

   Line 36: "collecting information paid by ...": should "information" be "rental prices"?

   Line 47: "this data sets ... limits": should be "these" and "limitations"

   Line 52: "from insurance. Its provides ..": should be "insurance companies" and "It provides"

   Line 55: "on local market" should be "on local market conditions" or "on local markets"

   Thank you very much for these suggestions. We sent this new version to an academic editing service.
