# Supplementary material for: Can big data increase our knowledge of local rental markets? A dataset on the rental sector in France
Source: PLoS One. 2022 Jan 27;17(1):e0260405. doi: 10.1371/journal.pone.0260405 (PMC8794157; doi:10.1371/journal.pone.0260405)
Supplement: Attachment — Submitted filename: letter_plosone__Copy_ (3)-3-5.pdf [file pone.0260405.s005.pdf]

2 Referee 1

1. Nc needs more explanation (e.g. at what frequency is it collected). In line with the Notes of Figure 2, I understand ns is the number of online ads per strata (e.g. properties in the market) and Nc is the number of units available. Does this mean all properties (including occupied) or only in the market (and if so, over which frequency)? In the former case, a ratio of 1 would mean the online data contains as many units in the market as available or put differently, it means all apartments in the strata are on the market. Let's assume the latter case. In this case I wonder how to interpret a ratio higher than 1? Does it mean there is more than one online add per available unit? In this case, I question the duplication filter. The authors need to be more specific. Based on the sentence in line 225, the overall exercise seems not like a measure of representativeness but turnover, as it is suggested the ratio increases over time. I therefore understand Nc is a local constant while ns is time dependent. It would be great to have a reference point from the literature as 1 seem a very high value (meaning every apartment is on average sold once within 2 years). Overall, the whole part is just very confusing raising strong doubts about it. In Figure 3 the ratio even goes up to 40 which I cannot explain. If you have any references for this methodology, I strongly advise to use them here and be more specific about the whole test.

   Thank you for raising point. We try now to make things clearer in the new version. The idea of this section is to assess the coverage of the dataset confronting it to the actual spatial distribution of the rental sector as observed in the census. The philioshopy of the test is inspired by the way the INSEE creates weights for their surveys using the margin adjustment method.We show that webscraping can provide some rental references for rural areas where the number of tenants is usually low. In these places, the ratio can be above 1 because we observe more ads than the actual number of tenants in the strata (usually, there are one or two tenants, and we observe 3 ads). This can be explained by the fact that some goods are vacants and thus on the market, we illustrate this in an appendix chart S2. Moreover, some places can also have a ratio higher than one when dwellings have a high turn-over and are put for rent several times per year. This can be the case in area where a lot of tenants are students or temporary worker in the tourism sector.

2. The data should have been truncated using rent per square meter directly, not by price and square meter separately. As a result, there are still outliers in the data (e.g., minimum rent per square meter: 0.2 Euro in Table 2). Please investigate.

   There are two parts in this paper. In the first part, the goal is to confront scraped data with alternate datasets. We truncate the data on the rent and not the rent per square meter in order to be fully comparable with the methodology of the local rental observatories as explained in the text.

Nevertheless, we fully agree that rental data should preferably be truncated on the rent per square meter. This is actually what we do in to two applications when estimating hedonic models following the guidelines of the INSEE. We truncate the dataset removing the first and the last centiles before running the regression. Finally, we now provide an additional table S.7 in appendix with the dataset truncated on the rent per square meter and show that the moments and distributions are not affected.

3. l.323: I am confused why the authors call it an index but estimate the model for each year individually (maybe this is just a wording problem)? Also, I don't understand how ln(c ref) is estimated. Technically, I understand that the local constant is seen as the index here, which would be in line with the literature. However, how is the logarithm applied or why is it assumed that the estimated constant is actually the logarithm. Please provide more details on the model derivation or provide some references to studies using a similar estimation. Interestingly, the authors later retransform the logarithms. Why not using levels directly then? Also, at which point is the calculation adjusted for property size or is the estimation on a per-square meter base? I think this section requires some rework and more explanations.

   This might be a wording problem. In line with the literature we estimate spatial indices. We apply the logarithm following the literature and provide additional references on the method. Our specifications are standard; hedonic models are usually performed using logs. The dependent variable is indeed the rent per square meter.

4. There are no units in Table 6. E.g. taking Paris as an example, I don't understand what 16.41 is. If this is in Euro, I assume it is per square meter per month, which would contradict the average in l. 427 though. This would raise the questions about the timely difference between rents and prices (rents would need to be adjusted for year or is this the monthly rent-price ratio?). In Figure 5, which is not linked to the text, it is indicated that the unit is percent.

   We updated the table and its footnote.

5. The used tense is constantly changing (present, past, etc.)
   We homogenised the tense used.

6. L.190 This is confusing. The authors say that they use public APIs to get the data but then state that they use HTML code to receive information. I am not aware of any API that provides HTML code. Does this mean the authors used the API and scrapped the website (double work so to say). In this case I wonder how much data could be generate by one or the other.

   This was confusing indeed, we corrected it

7. Table 1: It seems like online ads are not representing the full spectrum of the rental market. Social housing seems to be excluded. Does this have implications on the estimations of free market rents for social units. Curious to hear the authors opinion.

   Our dataset concerns the private rental sector that's why we restrict it to this sector. Social housing tenants do not look for flats in France, they have to register on a waiting list, the flats are then proposed to them by a commission. There is no choice based letting in France. Of course, our estimates are in partial equilibrium and do not answer the question "what would be the market rent of these dwellings if they were all suddenly liberalized and offered on the market ?". Each subsidy is estimated marginally (i.e. keeping the other social dwellings in the controlled sector).

8. L.207 This is unclear and I am not sure if "crossing" is the right word here.

   We try to make things clearer following your recommendations in 1

9. L. 221 vs. l.222 these sentences somehow contradict each other, maybe clarify.

   We clarified the mechanisms.

10. Table 3: What is a single unit or more specific what is the difference to 1 room? By single unit we meant a house by opposition to apartment. But this is indeed a mistake. We corrected it.

11. Equation (1) what is the unit of "a"? If it is Strata, I don't understand how area fixed effects can be used (perfect collinearity)? Please elaborate a bit more and present the degrees of freedom.

    a is the index of the area. There is no problem of colinearity. In the most demanding specification (columns 3 and 6), we include pair fixed effects. This is very standard in the literature, in particular in papers using matching methods. When pairs fixed effect are included, the degrees of Freedom remain high (Df=N-N/2-1).

12. Figure 4: It also seems that the relationship is not fully 45 degrees as it diverges for higher priced areas (ca. above 20 median rent), indicating that online ads are higher for these areas (observations). This is what is tested formally in Table S1 in the appendix. No particular bias appears for expansive markets.

13. l.362 the referenced paper is not by the same authors, so I would change the wording as readers might want to check the companion study. This was a typo. We corrected it.

14. l. 470 please elaborate on this conclusion

    This is discussed in l.366-l.371.
